# Clinico-genetic findings in 509 frontotemporal dementia patients

**DOI:** 10.1038/s41380-021-01271-2

**Published:** 2021-09-24

**Authors:** Matias Wagner, Georg Lorenz, Alexander E. Volk, Theresa Brunet, Dieter Edbauer, Riccardo Berutti, Chen Zhao, Sarah Anderl-Straub, Lars Bertram, Adrian Danek, Marcus Deschauer, Veronika Dill, Klaus Fassbender, Klaus Fliessbach, Katharina S. Götze, Holger Jahn, Johannes Kornhuber, Bernhard Landwehrmeyer, Martin Lauer, Hellmuth Obrig, Johannes Prudlo, Anja Schneider, Matthias L. Schroeter, Ingo Uttner, Ruth Vukovich, Jens Wiltfang, Andrea S. Winkler, Qihui Zhou, Albert C. Ludolph, The German FTLD consortium, Konrad Oexle, Markus Otto, Janine Diehl-Schmid, Juliane Winkelmann

**Affiliations:** 1 Institut für Neurogenomik, Helmholtz Zentrum München, Deutsches Forschungszentrum für Gesundheit und Umwelt (GmbH), Neuherberg, Germany; 2 Institute of Human Genetics, Technical University München, Munich, Germany; 3 Institute of Human Genetics, Helmholtz Zentrum München, Deutsches Forschungszentrum für Gesundheit und Umwelt (GmbH), Neuherberg, Germany; 4 Department of Nephrology, Klinikum rechts der Isar, Technical University Munich, Munich, Germany; 5 Institute of Human Genetics, University Medical Center Hamburg-Eppendorf, Hamburg, Germany; 6 German Center for Neurodegenerative Diseases (DZNE), Munich, Munich, Germany; 7 Munich Cluster of Systems Neurology (SyNergy), Munich, Germany; 8 Department of Neurology, University of Ulm, Ulm, Germany; 9 Lübeck Interdisciplinary Platform for Genome Analytics (LIGA), Institutes of Neurogenetics and Cardiogenetics, University of Lübeck, Lübeck, Germany; 10 Neurologische Klinik und Poliklinik, Ludwig-Maximilians-Universität, Munich, Germany; 11 Department of Neurology, Technische Universität München, School of Medicine, Munich, Germany; 12 Clinic and Policlinic for Internal Medicine III, Technical University Munich, School of Medicine, Munich, Germany; 13 Department of Neurology, Saarland University Medical Center, Homburg, Germany; 14 Department of Neurodegenerative Diseases and Geriatric Psychiatry, University Bonn, Bonn, Germany; 15 German Center for Neurodegenerative Diseases (DZNE), Bonn, Germany; 16 Clinic for Psychiatry and Psychotherapy, University Medical Center Hamburg-Eppendorf, Hamburg, Germany; 17 Department of Psychiatry and Psychotherapy, Universitätsklinikum Erlangen, and Friedrich-Alexander-Universität Erlangen-Nürnberg, Erlangen, Germany; 18 Department of Psychiatry, Psychosomatic Medicine and Psychotherapy, University of Würzburg, Würzburg, Germany; 19 Department of Neurology, Max Planck Institute for Human Cognitive and Brain Sciences, Leipzig, Germany; 20 Clinic for Cognitive Neurology, University Hospital Leipzig, Leipzig, Germany; 21 Department of Neurology, Rostock University Medical Center, German Center for Neurodegenerative Diseases (DZNE), Rostock, Germany; 22 Department of Psychiatry and Psychotherapy, University Medical Center Goettingen (UMG), Georg-August University, Goettingen, Germany; 23 German Center for Neurodegenerative Diseases (DZNE), Goettingen, Germany; 24 Neurosciences and Signaling Group, Institute of Biomedicine (iBiMED), Department of Medical Sciences, University of Aveiro, Aveiro, Portugal; 25 Centre for Global Health, Institute of Health and Society, University of Oslo, Oslo, Norway; 26 German Center for Neurodegenerative Diseases (DZNE), Ulm, Oberer Eselsberg, Ulm, Germany; 27 Department of Neurology, Martin Luther University Halle-Wittenberg, Halle, Germany; 28 School of Medicine, Department of Psychiatry and Psychotherapy, Technical University of Munich, Munich, Germany; 29 Chair of Neurogenetics, Technical University of Munich, Munich, Germany

**Keywords:** Psychiatric disorders, Genetics, Predictive markers

## Abstract

Frontotemporal dementia (FTD) is a clinically and genetically heterogeneous disorder. To which extent genetic aberrations dictate clinical presentation remains elusive. We investigated the spectrum of genetic causes and assessed the genotype-driven differences in biomarker profiles, disease severity and clinical manifestation by recruiting 509 FTD patients from different centers of the German FTLD consortium where individuals were clinically assessed including biomarker analysis. Exome sequencing as well as *C9orf72* repeat analysis were performed in all patients. These genetic analyses resulted in a diagnostic yield of 18.1%. Pathogenic variants in *C9orf72* (*n* = 47), *GRN* (*n* = 26), *MAPT* (*n* = 11), *TBK1* (*n* = 5), *FUS* (*n* = 1)*, TARDBP* (*n* = 1), and *CTSF* (*n* = 1) were identified across all clinical subtypes of FTD. *TBK1*-associated FTD was frequent accounting for 5.4% of solved cases. Detection of a homozygous missense variant verified *CTSF* as an FTD gene. *ABCA7* was identified as a candidate gene for monogenic FTD. The distribution of APOE alleles did not differ significantly between FTD patients and the average population. Male sex was weakly associated with clinical manifestation of the behavioral variant of FTD. Age of onset was lowest in *MAPT* patients. Further, high CSF neurofilament light chain levels were found to be related to *GRN*-associated FTD. Our study provides large-scale retrospective clinico-genetic data such as on disease manifestation and progression of FTD. These data will be relevant for counseling patients and their families.

## Introduction

Frontotemporal dementia (FTD) is a clinically heterogeneous neurodegenerative disorder with a hereditary component. FTD is characterized by progressive atrophy of the frontal and temporal lobes. The heterogeneous spectrum can be classified according to (1) the clinical presentation, (2) genetic diagnosis, and (3) histopathological findings [1].

Symptoms usually include deterioration of cognitive skills in combination with either predominant abnormalities of behavior and personality (behavioral variant of FTD, bvFTD) or language (primary progressive aphasia, PPA). The latter is subcategorized in non-fluent variant (nfv) PPA, logopenic variant (lv) PPA, and semantic variant (sv) PPA [2]. FTD is accompanied by motor neuron disease (MND) in about 15% of patients [3, 4].

Patients with a causative genetic variant can be classified further according to the respective FTD gene involved. Only about one fifth of all cases have a monogenic cause [5]. Of these, 80% are explained by variants in *C9orf72, MAPT* or *GRN*.

FTD can be classified neuropathologically based on the predominant protein abnormalities. Currently, four molecular subgroups of frontotemporal lobar degeneration (FTLD) have been established with FTLD-Tau being characterized by Tau depositions, FTLD-TDP by TDP-43 aggregates, and FTLD-FET by protein abnormalities of the FET (FUS, EWS, and TAF15) protein family. Rare cases with aggregates that only stain positive for ubiquitin are classified in the FTLD-UPS subgroup named after the ubiquitin/proteasome system [6].

Despite a high degree of correlation between neuropathological classification and genetic cause, it is challenging to predict the genetic diagnosis based on the clinical findings. This task is further complicated by a number of conditions with significant phenotypic overlap [7].

As the genetic cause and the molecular pathogenesis will be important for therapeutic decisions, there have been significant efforts to find molecular biomarkers that assist in disease categorization during the patient’s lifetime. The measurement of serum or cerebrospinal fluid (CSF) progranulin (PGRN) levels has been implemented already in routine clinical practice with low levels predicting the diagnosis of *GRN*-related FTD [8]. Other potential biomarkers include neurofilament light chains (NfL) which predict survival, as well as microRNAs miR-204-5p and miR-632 which are associated with the clinical manifestation of FTD [9, 10].

In the present study, we aimed to better define the clinico-genetic spectrum of FTD using exome sequencing (ES) and *C9orf72* repeat analysis in 509 patients. Thereby, we demonstrate the importance of genetic data as a biomarker.

## Methods

### Cohort

A total of 509 unrelated cases were recruited via the German FTLD consortium. Among these, 162 cases were referred by the Center for Cognitive Disorders and Cognitive Rehabilitation, Munich, Germany and 347 cases were enrolled via further FTLD consortium dementia clinics from neurologic and psychiatric university hospitals in Munich, Ulm, Würzburg, Bonn, Erlangen, Göttingen, Hamburg, Homburg, Rostock, and Leipzig using a common standardized database. The study protocol received approval by all local ethics committees and all subjects or their legal representatives provided written informed consent. All patients included in this study met the 2011 diagnostic criteria for probable or definite bvFTD or PPA [2, 11].

### Clinical and neurochemical testing

All patients underwent detailed neuropsychological examination. Disease severity was assessed using the FTLD‐specific Clinical Dementia Rating (FTLD‐CDR) score [12]. CSF was collected to determine Tau, phosphorylated Tau (p-Tau), Amyloid-beta (Aβ) (1-42), as well as CSF PGRN, phosphorylated neurofilament heavy chain (pNfH), and NfL levels. Data on these biomarkers was available in 226, 180, 224, 173, 187, and 166 individuals, respectively. In addition, blood samples were collected to measure serum NfL and serum PGRN as previously described [13, 14]. Data on these biomarkers was available in 201 and 75 subjects, respectively.

Family history was assessed using the query, “Are there any neuropsychiatric disorders in your family up to the grand-parental generation?”. Neuropsychiatric disorders included dementia, amyotrophic lateral sclerosis (ALS), Parkinson syndromes, psychosis, depression, and suicide. Information on the family history was available for a total of 385 cases.

### Genetic testing

*C9orf72* testing as well as exome sequencing was performed in all individuals. To test for a hexanucleotide repeat expansion in *C9orf72* PCR‐based screening methods were used [15]. Exome sequencing (ES) was performed as previously described [16]. Sure Select Human All Exon kits V5 and V6 (Agilent, Santa Clara, CA, USA) were used for exome enrichment. Paired end sequencing was performed on HiSeq2500 or HiSeq4000 systems (Illumina, San Diego, CA, USA) to an average read depth of at least 100x. Reads were aligned to the UCSC human reference assembly (hg19) with Burrows-Wheeler algorithm (BWA v.0.7.5a). Single-nucleotide variants (SNVs) as well as small insertions and deletions were detected with SAMtools v.0.1.19. Only variants with a minor allele frequency <0.1% were considered in the analysis.

All samples underwent a three-step analysis: in the first step, variants in FTD genes *GRN*, *MAPT*, *TBK1*, *TARDBP*, *VCP*, *SQSTM1*, *CHMP2B*, and *TIA1* were considered and classified according to the guidelines of the American College of Medical Genetics [17]. In the second step, variants in the genes *APP*, *PSEN1*, and *PSEN2* were screened for due to their association with monogenic Alzheimer’s disease (AD), a disease that may phenocopy FTD. In the third step all heterozygous variants with an allele frequency <0.01% as well as homozygous and potentially compound heterozygous variants were considered. For assessment of the ApoE status, the three alleles ApoE2, ApoE3 and ApoE4 were determined according to the presence of variants rs7412 and rs429358 in the ES data.

Genome sequencing was performed using TruSeq DNA PCR-free library preparation kits and a HiSeq4000 system (both Illumina, San Diego, CA, USA) for sequencing as 2 × 150 bp sequencing reads to an average coverage of 30x.

Cases were considered to have a definite genetic diagnosis if a variant was identified which was classified as pathogenic or likely pathogenic. All other cases were considered as genetically unsolved throughout the manuscript.

### Statistical analysis

Rare variant burden testing was done by assessing the gene-wise enrichment of rare (minor allele frequency ≤0.1%) coding loss-of-function variants in 509 FTD cases in comparison to 12,126 in-house controls. These controls, however, are not age-matched. The *p* value was calculated by the Fisher’s exact test. To account for multiple testing, we used Bonferroni correction and set the significance threshold at 2.5 × 10^−6^ (corresponding to 20,000 genes/hypotheses).

Statistical analyses were performed using IBM SPSS Statistics 23 and R 3.6.2. Missing values were excluded from analyses. We present mean ± standard deviation (SD), median and [interquartile-range] or counts and (% of superset) for normally distributed data, non-normally distributed metric and ordinal variables or nominal data, respectively. *P* < 0.05 were considered statistically significant, except for in the rare variant burden testing.

## Results

### Baseline demographics

Baseline demographic details are summarized in Table 1. Of 509 unrelated cases, 45% had a clinical diagnosis of bvFTD. 5% of PPA patients could not be assigned to a specific subgroup (not classified PPA, ncPPA). PPA and FTD patients with additional signs of MND were classified as FTD/MND and might be underrepresented with 7% as ALS clinics were not involved in recruiting cases. 13% of cases had oligosymptomatic FTD or had features of two or more clinical subtypes and could therefore not be definitely assigned to a specific subgroup (not classified FTD, ncFTD). 55% (280/509) of all cases were male, whereas 61% (139/228) of patients with a clinical diagnosis of bvFTD were male. Thus, male patients appeared to be slightly enriched in the bvFTD group (*P* = 0.02, chi-square test, bvFTD versus other phenotypes).

**Table 1:** Baseline characteristics of the FTD cohort.

Clinical diagnosis	Overall	bvFTD	FTD/MND	nfvPPA	svPPA	lvPPA	ncPPA	ncFTD
Number of cases	509	228 (45%)	38 (7%)	57 (11%)	62 (12%)	33 (6%)	23 (5%)	68 (13%)
Sex distribution								
Male	280 (55%)	139 (61%)	20 (53%)	24 (42%)	29 (47%)	18 (55%)	10 (43%)	40 (59%)
Female	229 (45%)	89 (39%)	18 (47%)	33 (58%)	33 (53%)	15 (45%)	13 (57%)	28 (41%)
Age of onset								
Age of onset, yrMedian [IQR]	61.0 [55.0–68.0]	58.0 [51.0– 66.0]	61.0 [55.0–67.0]	66.0 [60.0–71.0]	61.5 [56.3–66.8]	65.0 [60.0–68.0]	65.0 [57.0–72.0]	61.0 [54.0–67.0]
Age at examination, yrMedian [IQR]	64.0 [58.0–71.0]	61.5 [56.0–69.0]	63.0 [58.0–69.3]	69.0 [61.3–73.0]	64.0 [60.0–71.0]	68.0 [64.5–72.0]	69.0 [61.0–74.0]	65.0 [58.0–69.0]
Disease duration, median [IQR]	2.0 [1.0–4.0]	3.0 [1.0–5.0]	1.0 [1.0–3.0]	2.0 [1.0–3.0]	3.0 [2.0–5.0]	2.0 [1.0–5.5]	2.0 [1.0–4.0]	2.0 [1.0–3.0]
FTLD-CDR score, mean/SD	7.4/6.0	8.9/6.0	7.1/5.0	5.1/3.3	6.2/4.3	4.9/3.4	7.8/4.7	7.4/6.0

*yr* years, *SD* standard deviation, *CDR* clinical dementia rating, *nc* not classified.

### Genotype distribution

We were able to establish a genetic diagnosis in 92 cases (18.1%), similar to previously published rates [18] on genetic diagnoses in FTD. Of these, 47 cases (51%) had a repeat expansion in *C9orf72*. ES led to genetic diagnoses with variants in *GRN* in 26 cases (28%), *MAPT* in 11 cases (12%), *TBK1* in 5 cases (5%), and *FUS*, *TARDBP* and *CTSF* in 1 case each (together 3%). 13% (6/45) of variants have not been observed so far and can be considered as novel. No dual diagnoses were made. Identified variants and their pathogenicity are listed in Supplementary Table 1. At the time of enrollment 15/45 (33%) individuals who finally received a genetic diagnosis already had a known mutation, whereas 30/45 (67%) were genetically solved within the study.

**Fig. 1:**
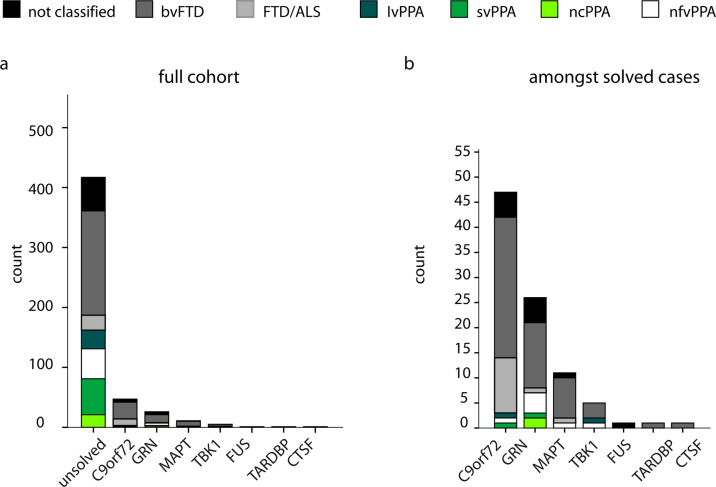
Clinical distribution for genetic subgroups of FTD. The figure shows the distribution of clinical subtypes of FTD according to their genetic diagnosis (see **a** for the distribution within the full cohort and **b** within the solved cases). There was no significant difference in the distribution of clinical diagnoses between the genetic subgroups.

We further assessed whether the genetic diagnosis influences the clinical presentation. Pathogenic variants in *C9orf72*, *GRN*, and *MAPT* could be identified in most clinical subgroups: bvFTD, FTD/MND, and PPA (Fig. 1). There was no overall significant difference in the distribution of all clinical diagnoses between all genetic subgroups in our cohort (*P* = 0.059, chi-square test). Yet, when analysing for every gene separately, a pathogenic repeat expansion in *C9orf72* was significantly associated with the clinical diagnosis of FTD-ALS (*P* < 0.001, Bonferoni corrected *p* < 0.0037). *P* values for all tests are displayed in Supplementary Table 2.

Interestingly, none of the 5 patients with pathogenic variants in the well-established ALS-gene *TBK1* had motor neuron symptoms [19].

In order to evaluate if our cohort is enriched with AD patients, we assessed the frequency of the ApoE alleles given that ApoE4 is associated with an increased risk of Alzheimer’s disease [20]. Frequencies (Supplementary Table 3) of the respective ApoE alleles in our FTD cohort did not differ significantly from published data nor from the genome aggregation database (gnomAD) frequencies (*P* = 0.61, Chi-square test) [21, 22].

### Family history

Information on the family history was available for 385 cases. Across these, 24.7% (95/385) reported a positive family history for neuropsychiatric disorders. 42.1% (40/95) of individuals with a positive family history and 12.4% (36/290) with a negative family history could be genetically diagnosed (see Supplementary Figs. 1 and 2). The diagnostic yield was highly correlated with the number of affected family members (*r* = 0.96, Pearson’s correlation coefficient) and increased to 75% (12/16) when only considering cases with three or more affected family members. Assessing the medical records of four individuals with three or more affected family members revealed that in three pedigrees, affected relatives had a diagnosis of Alzheimer or Parkinson disease. Only one pedigree showed both FTD and ALS diagnoses in first- and second degree relatives (see Additional genetic findings). In addition, we assessed the frequency of a positive family history among individuals with a pathogenic repeat expansion in *C9orf72* or a variant in *GRN* and *MAPT*. Interestingly, 47% (22/47), 50% (13/26), and 73% (8/11) had negative family histories, respectively (Supplementary Fig. 3).

### Age of onset

**Fig. 2:**
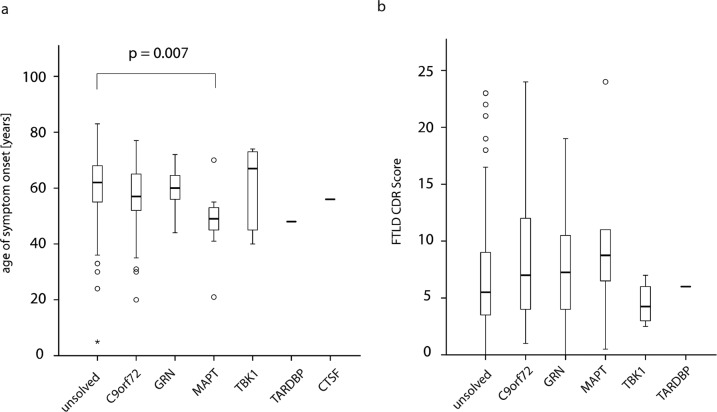
Age of onset and disease severity compared between the genetic subgroups. Individuals without a definite genetic diagnosis are labeled as “unsolved” (**a**) The median age of onset was compared between the genetic subgroups with *MAPT* patients having the earliest age of onset (*P* = 0.007, post-hoc test). **b** The severity of FTD at the first presentation as assessed using the FTLD-CDR score was highest (most severe) in patients with the pathogenic variants in *MAPT* whereas cases with pathogenic *TBK1* variants were least severely affected, however, differences were not significant Outliers are depicted as separate dots with their cohort ID.

The median age of onset across all individuals was 61.0 [IQR: 55.0–68.0] years (Fig. 2A). We observed a significant difference in age of onset across the genetic subgroups (*P* = 0.001, Kruskal–Wallis-test). *MAPT*-patients had the earliest clinical symptoms (49.0 years [IQR: 44.0–53.5 years]) followed by *C9orf72* (57.0 years [IQR: 50.0–65.0 years]). On median, *TBK1*-patients had the latest age of onset but also the largest variability, implying a relatively stronger influence of modifying factors (67.0 years [IQR: 42.5–73.5 years]). *GRN*-patients had initial symptoms at a median age of 60.0 [IQR: 56.0–64.8] years.

### Assessment of severity

Higher FTLD-CDR scores are associated with a more severe clinical presentation. Across genetic diagnoses, no significant difference in disease severity was observed (*P* = 0.3). Results are displayed in Fig. 2B. In addition, the rate of decline was not significantly different between the genetic subgroups (Supplementary Fig. 4).

### Serum and CSF biomarkers in FTD

**Fig. 3:**
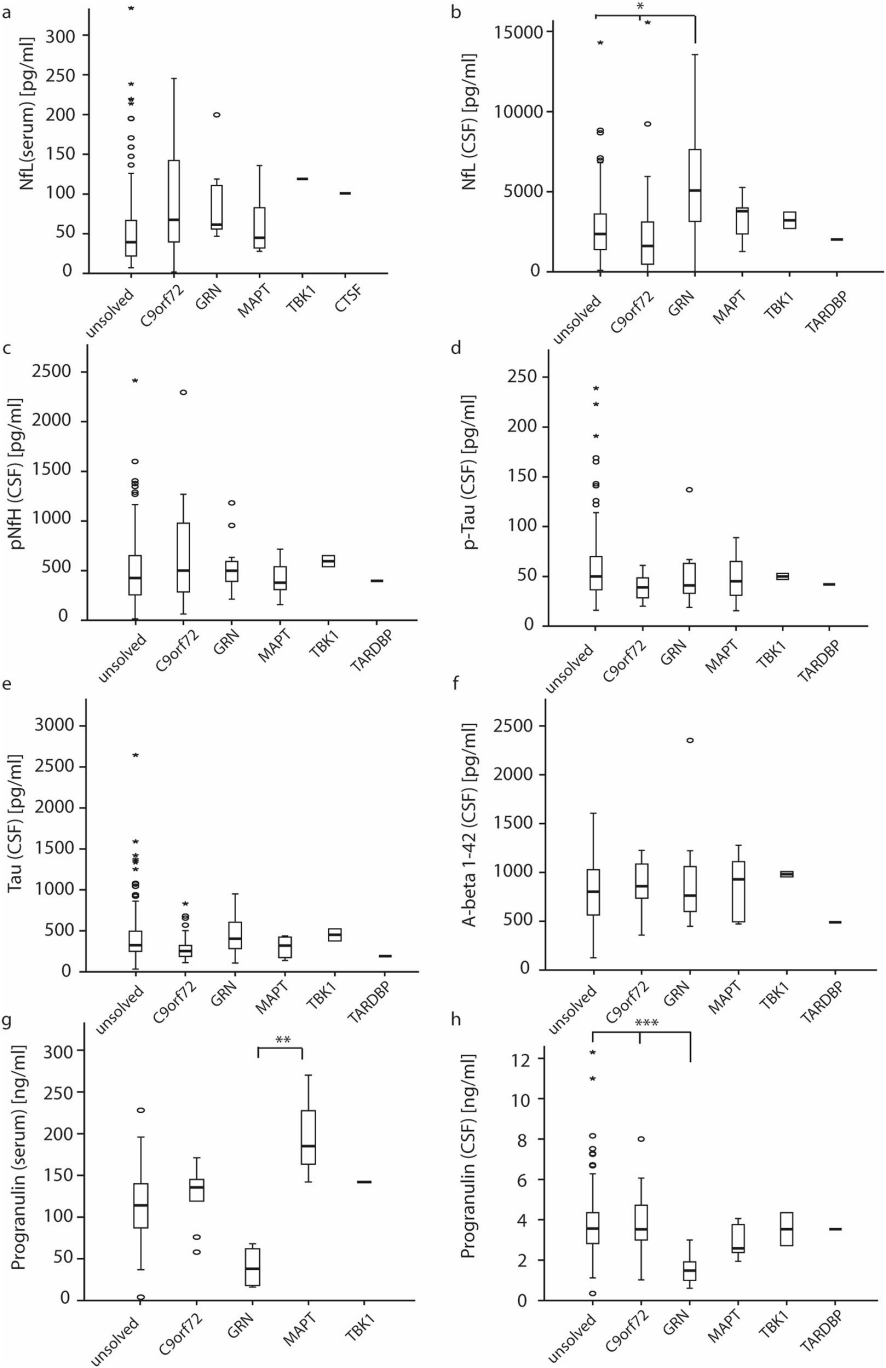
Comparison of neurochemical findings between the genetic subgroups. The figures depict the mean levels of biomarkers and the 95% confidence intervals. Outliers are plotted as separate dots. The figures show the differences between serum NfL (**a**), CSF NfL (**b**), pNfH (**c**), CSF p-Tau (**d**), CSF Tau (**e**), CSF A-beta 1-42 (**f**), serum progranulin (**g**) and CSF progranulin (**h**) levels between *C9orf72-*, *GRN-*, *MAPT-*, *TBK1-*, *TARDBP1-* and genetically unsolved patients. Only CSF NfL as well as serum and CSF progranulin levels were significantly different between subgroups. Significantly different levels between subgroups are highlighted using asterisks (post-hoc test, *P < 0.5, **P = 0.007, ***P < 0.001). Note, that patients with the clinical diagnosis of FTD/MND were omitted in figures depicting NfL levels as MND would cause elevated levels independent from the genetic diagnosis.

We next evaluated if serum and CSF biomarkers differ between the different genetic diagnoses or could help to predict them. There was no significant difference between the genetic subgroups for serum levels of pNfH, p-Tau, Tau, and Aβ1-42 (Kruskal–Wallis test for multiple unrelated samples, Fig. 3A, C–F). Interestingly, CSF pTau and Tau were comparable to genetically unsolved cases in individuals with a pathogenic variant in *MAPT*. Patients with pathogenic *GRN* variants had higher levels of CSF NfL (Fig. 3B, median = 5082 [IQR: 3084–7637] pg/mL) than the other solved or unsolved cases (2119 [IQR: 1470–3840] pg/mL, *P* = 0.011, Kruskal–Wallis test) [23, 24]. In addition, decreased levels of PGRN in serum (median 38.0 [IQR: 17–65] ng/mL) and CSF (median = 1.48 [IQR: 0.97–1.96] ng/mL) were found in comparison with the other groups (median 3.45 [IQR: 2.60–4.08] ng/mL, *P* < 0.001 for CSF and median 116 [IQR: 86–141] ng/mL, *P* = 0.008 for serum PRGN, Kruskal–Wallis test). Of note, three individuals in whom ES could not identify a pathogenic *GRN* variant, had low PRGN levels in either CSF (1.03 ng/mL in two cases) or serum (4.0 ng/mL), suggesting the genetic diagnosis of *GRN*-related FTD. We performed whole-genome sequencing to search for potentially pathogenic noncoding or structural variants in *GRN* in these three cases. However, no rare variant could be identified implying that short-read sequencing failed to detect the pathogenic variants or that there are other—so far unidentified—causes of low PRGN levels.

**Fig. 4:**
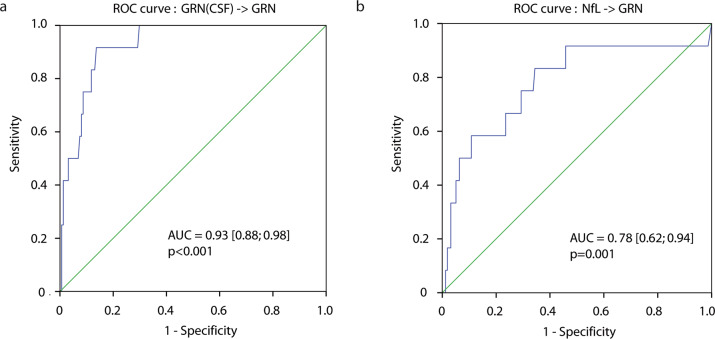
ROC analysis of CSF progranulin and CSF NfL to predict the presence of a pathogenic *GRN* variant. **a** depicts the ROC analysis of CSF progranulin to predict *GRN*-associated FTD with a calculated optimum cutoff at 2.04 ng/mL (Sensitivity = 0.886, 1-Specificity = 0.000, Youden’s J = 0.886). **b** used serum NfL as a marker to predict the presence of a pathogenic variant in *GRN*. The AUC was 0.78 [0.62–0.94], *P* = 0.001 and the calculated optimum cutoff was 3258 ng/mL (Sensitivity = 0.889, 1-Specificity = 0.297, Youden’s J = 0.592).

In order to assess the clinical utility of assaying serum and CSF PGRN and CSF NfL, we performed a receiver operating characteristic (ROC) curve analysis of the cut-off-dependent accuracy (that is, relation between sensitivity and specificity) in predicting the presence of a pathogenic *GRN* variant as displayed by the area under the curve (AUC) which has an optimum of 1. ROC analysis for serum PGRN was underpowered and is not displayed. Based on 12 *GRN* cases where CSF PGRN was available, the AUC was 0.93 (95% CI: 0.88–0.98, *P* < 0.001, Fig. 4A). The AUC (*GRN* cases vs. other patients) for CSF NfL was 0.78 (95% CI 0.62–0.94, *P* = 0.001, Fig. 4B). CSF NfL and CSF PGRN levels were not correlated in the overall cohort (Pearson *r* = −0.09, *P* = 0.345, *n* = 158) but a non-significant trend was observed in *GRN* patients (Pearson *r* = −0.41, *P* = 0.18, *n* = 12). CSF NfL did not significantly correlate with FTLD-CDR scores (rho = −0.14, *P* = 0.7, *n* = 10) or disease duration (rho = 0.25, *P* = 0.45, *n* = 11) in the GRN subgroup.

### Additional genetic findings

Beyond from the detection of pathogenic variants in established FTD genes, we identified a homozygous missense variant p.(Ile416Thr) in *CTSF* in a female patient from a consanguineous family. The variant was classified as “likely pathogenic” as it was previously described in a patient with FTD [18, 25]. The patient had symptoms since the age of 56 years and presented at the age of 60 years when she was diagnosed with bvFTD.

In a female patient without a genetic diagnosis who was seen at the age of 74 years, 5 years after onset, svPPA was diagnosed. ES identified a homozygous 7-bp deletion in *ABCA7* (NM_019112.3): c.2126_2132del, p.(Glu709AlafsTer86). The variant is predicted to induce a frameshift and a complete loss-of-function. Genome-wide association studies (GWAS) have identified the *ABCA7* locus to be associated with AD, and heterozygous loss-of-function variants in *ABCA7* including p.(Glu709AlafsTer86) have been shown to confer risk of AD [26–28]. To our knowledge, a link between *ABCA7* and FTD has not been established before.

In one case with four affected family members diagnosed with FTD and/or ALS, we identified a heterozygous in-frame insertion in *RBM33* (NM_053043.2): c.1876_1877insAGCCCC, p.(His625_Pro626insGlnPro). This gene was prioritized because it contains a prion-like domain and because of properties shared among proteins associated with ALS and FTD which allow them to self-associate and form protein aggregation in a disease state [29]. Samples from family members were not available for segregation analyses.

*TIA1* has recently been postulated as a novel ALS and FTD gene [30]. However, resequencing studies were unable to replicate this finding [31, 32]. We found 3 rare (MAF < 0.1%) *TIA1* missense variants in the 417 unsolved FTD cases (NM_022037.2: c.1045 G>A, p.(Val349Met); c.698 G>A, p.(Arg233Gln), and c.401 A>C, p.(Lys134Thr)). However, 12,126 control exomes revealed 249 rare coding missense variants of *TIA1* indicating a depletion rather than an enrichment in our cases (*P* = 0.051, Fisher’s exact test). Our study, therefore, does not indicate a causal role for *TIA1* variants in FTD.

A recent study demonstrated rare coding and noncoding variants in *TET2* to be associated with different forms of neurodegenerative disease including FTD [33]. The authors described an enrichment of rare variants in *TET2* in the discovery set of a combined early-onset Alzheimer’s disease (EOAD) and FTD cohort, with an odds ratio (OR) of 28.9 (4.5–1200); *P* = 4.6 × 10^−8^ [33]. Our rare variant burden test (Supplementary Fig. 5) identified an enrichment for rare loss-of-function variants in *GRN* and indeed, with the second strongest enrichment after *GRN* (*P* = 2.2 × 10^−21^), we observed a nominally significant enrichment of variants in *TET2* in the FTD cohort (*P* = 6.4 × 10^−6^). In total, we identified *TET2* loss-of-function variants in 8 individuals in the FTD cohort and in 18 control individuals (Supplementary Tables 4 and 5). Moreover, we noticed an unusual variant allele fraction, i.e., percentage of NGS reads indicating a mutant allele in 7/8 individuals from the FTD cohort, in whom it ranged from 9% to 13%, suggestive of somatic mosaicism.

## Discussion

Genetics has much improved our understanding of FTD. However, comprehensive sequencing studies that have tried to further unravel the genetic spectrum of FTD have been lacking in clinical information [18]. We provide an aggregate of clinical, neurochemical, and genetic findings in the largest FTD cohort to date.

A first finding in our cohort was a male predominance among patients with a clinical diagnosis of bvFTD. Males accounted for 61% of all bvFTD cases. Unequal sex distribution has been reported in FTD, regardless of the clinical subgroup [34–36]. Moreover, a sex difference has been observed for the prevalence of *GRN*-related as compared to *C9orf72*- and *MAPT*-related FTD, which was interpreted as sex differences in penetrance among the subgroups [37]. Our study again highlights the influence of sex on the clinical expression of FTD with male sex predisposing for the development of a bvFTD subtype.

We did not find any significant correlation between the clinical diagnoses and the mutated genes, only individuals with FTD/MND were enriched in the *C9orf72*-subgroup. Among the genetic subgroups with five or more patients, only the *TBK1* group did not comprise any case with motor neuron symptoms, which was surprising because *TBK1* was initially published as an ALS gene and was only later associated with FTD [19, 38]. However, the absence of FTD/MND among our *TBK1* patients was not significant, and indeed, *TBK1* patients with FTD/MND have been reported before [39]. Hence, our findings suggest the rule “any gene—any clinical FTD subgroup” (Fig. 1) and is thereby in keeping with previous studies [40]. However, even larger prospective studies as well as meta-analyses are required to better understand if and how underlying genetic defects influence the clinical presentation.

The ascertainment of the 509 unrelated patients was not biased for monogenic cause. Therefore, the 92 genetic diagnoses represent a good estimate of the presently possible diagnostic yield (18.1%), confirming previous findings [18]. Among the solved cases, the most frequent genetic diagnosis was due to pathogenic variants in *C9orf72*, followed by *GRN* and *MAPT*. We believe that our data on the frequencies of genetic diagnoses provides a robust estimation of the frequencies in Europe.

Regarding rare genetic FTD subtypes, we report the third case of *CTSF*-associated FTD [18, 25]. Biallelic variants in *CTSF* were initially described as causative for neuronal ceroid lipofuscinosis 13 [41]. However, shortly thereafter, pathogenic biallelic variants in *CTSF* were described in patients with early-onset AD or FTD [18, 25, 42]. We believe that loss of CTSF causes a neurodegenerative disorder with a broad phenotypic spectrum that primarily presents with dementia. As variants in *CTSF* can now be regarded as an established monogenic cause of FTD, we recommend to include this gene in routine genetic testing.

The large number of undiagnosed patients in our cohort (81.9%) raises the question of unresolved genetic variation and environmental factors predisposing to FTD [43]. We expect that a large proportion of FTD cases have polygenic/multifactorial causes, including variants at the risk loci identified by GWAS [44]. Moreover, we expect cases to have constitutional monogenic causes, including cases with VUS reclassified as pathogenic variants, copy number variation, non-coding variants, and repeat expansions detectable only by specific methods such as (long-read) WGS. Heterozygous loss-of-function variants in *ABCA7* have been associated with AD [28]. We observed a homozygous loss-of-function variant in *ABCA7* in an FTD patient, further indicating an overlap in the genetic architecture of AD and FTD [45]. However, the common AD risk allele ApoE4 which has been reported to be increased in FTD did not show a significant enrichment in our cases (*P* = 0.61) [46]. This finding also excludes substantial AD contamination of our FTD cohort.

We were also unable to unambiguously replicate recent findings of an association of rare loss-of-function variants in *TET2* with FTD [33]. We performed burden testing and observed nominally significant enrichment of loss-of-function variants in *TET2*. However, a very low variant allele balance indicated clonal hematopoiesis. Interestingly, *TET2* is the second most frequent gene to be associated with clonal hematopoiesis of indeterminate potential (CHIP) [47]. CHIP is an age-related process of hematopoietic progenitor cells caused by acquired somatic mutations in genes associated with myeloid malignancies. With a prevalence of 10–20% in those older than 70 years, clonal hematopoiesis is common in the elderly population [48]. Thus, we believe that the enrichment of variation in *TET2* in our cohort and the EOAD and FTD cohorts published by Cochran et al. is confounded by the probands’ age as the latter is associated with increased CH. Recent studies, however, have suggested that CHIP contributes to cardiac dysfunction and atherosclerosis via activation of inflammatory signaling pathways [49]. CHIP might also pose a risk factor for neurodegenerative disorders as neuroinflammation has been implicated in their pathogenesis [50].

We also assessed differences in the age of onset and the severity of disease progression across the common genetic subtypes of FTD. *MAPT* and *C9orf72* patients had a relatively early age of onset, whereas age of onset was late in *TBK1* patients. In contrast, dementia severity at first presentation as measured with the FTLD-CDR did not differ significantly between the genetic subgroups.

Various serum and CSF biomarkers were compared between the genetic subgroups. β-amyloid (Aβ1-42), Tau, and phosphorylated Tau (p-Tau) only helped to differentiate between AD and FTD for the selection of cases (data not shown), even though some of the cases still had low Aβ1-42 and high Tau and p-Tau levels indicating AD comorbidity. Similarly to other tauopathies, such as progressive supranuclear palsy (PSP) and corticobasal degeneration (CBD), CSF Tau and p-Tau levels were not elevated in individuals with pathogenic variants in *MAPT* indicating that Tau levels do not serve as a biomarker for *MAPT*-associated FTD [51]. High NfL levels as well as low PGRN levels have been shown to be associated with the genetic diagnosis of *GRN*-FTD [23, 24, 52]. In keeping with these studies, low CSF PRGN and high CSF NfL levels predicted the presence of *GRN* variants. Measurement of PRGN is well established as a state marker for *GRN*-associated FTD, whereas NfL has only recently been introduced as a trait marker indicating the onset of the disease with an increase of levels 2–4 years before conversion [53, 54]. Interestingly, in patients with variants in *GRN*, levels continuously increased, whereas in other genetic forms of FTD, levels remained stable after conversion. As NfL constitutes a marker for neurodegeneration, a potential explanation would be progressive disease activity and neurodegeneration in patients with *GRN*-associated FTD. However, CSF NfL levels in *GRN* variant carriers were not significantly correlated with the disease duration and the severity, suggesting that the association of CSF NfL with the genetic diagnosis of *GRN*-associated FTD requires further explanation beyond the role as a state marker in FTD.

Our results support performing genetic testing in cases with a positive family history. Interestingly, the clinical diagnosis was a comparably weak predictor in our study group. Nevertheless, the diagnostic yield of 12.4% in cases with no affected family members indicates that genetic testing should be offered to all patients.

In summary, our large-scale cross-sectional study highlights the importance of genetic testing in FTD. We provide a framework of clinical and neurochemical differences between the genetic subgroups which provides guidance for genetic counseling and future large-scale studies on clinical, radiological, neurochemical, genetic, and post-mortem features of FTD.

Supplementary information is available at MP’s website.

## Supplementary information

Supplemental Material

## Data Availability

Pathogenic and likely pathogenic variants were submitted to ClinVar and are deposited with the following accession numbers: *GRN*: VCV000976698, VCV000807610, VCV000098130, VCV000098134, VCV000807611, VCV000807612, VCV000098246, VCV000203460, VCV000807613, VCV000098150, VCV000098152, VCV000203456, VCV000807426, VCV000098177. *MAPT*: VCV000098213, VCV000014245, VCV000098222, VCV000014262, VCV000665222, VCV000807628. *TBK1*: VCV000807704, VCV000807705, VCV000807706, VCV000807707, VCV000807508, VCV000813327. *FUS*: VCV000447355. *CTSF*: VCV000807589. *TARDBP*: VCV000021476.
