# Supplementary material for: Clinico-genetic findings in 509 frontotemporal dementia patients
Source: Mol Psychiatry. 2021 Sep 24;26(10):5824–32. doi: 10.1038/s41380-021-01271-2 (PMC8758482; doi:10.1038/s41380-021-01271-2)
Supplement: Supplementary file 1 — Supplemental Material [file 41380_2021_1271_MOESM1_ESM.docx]

**Clinico-genetic findings in 509 frontotemporal dementia patients.**

**Supplementary Information**

**Supplementary Figures**

**
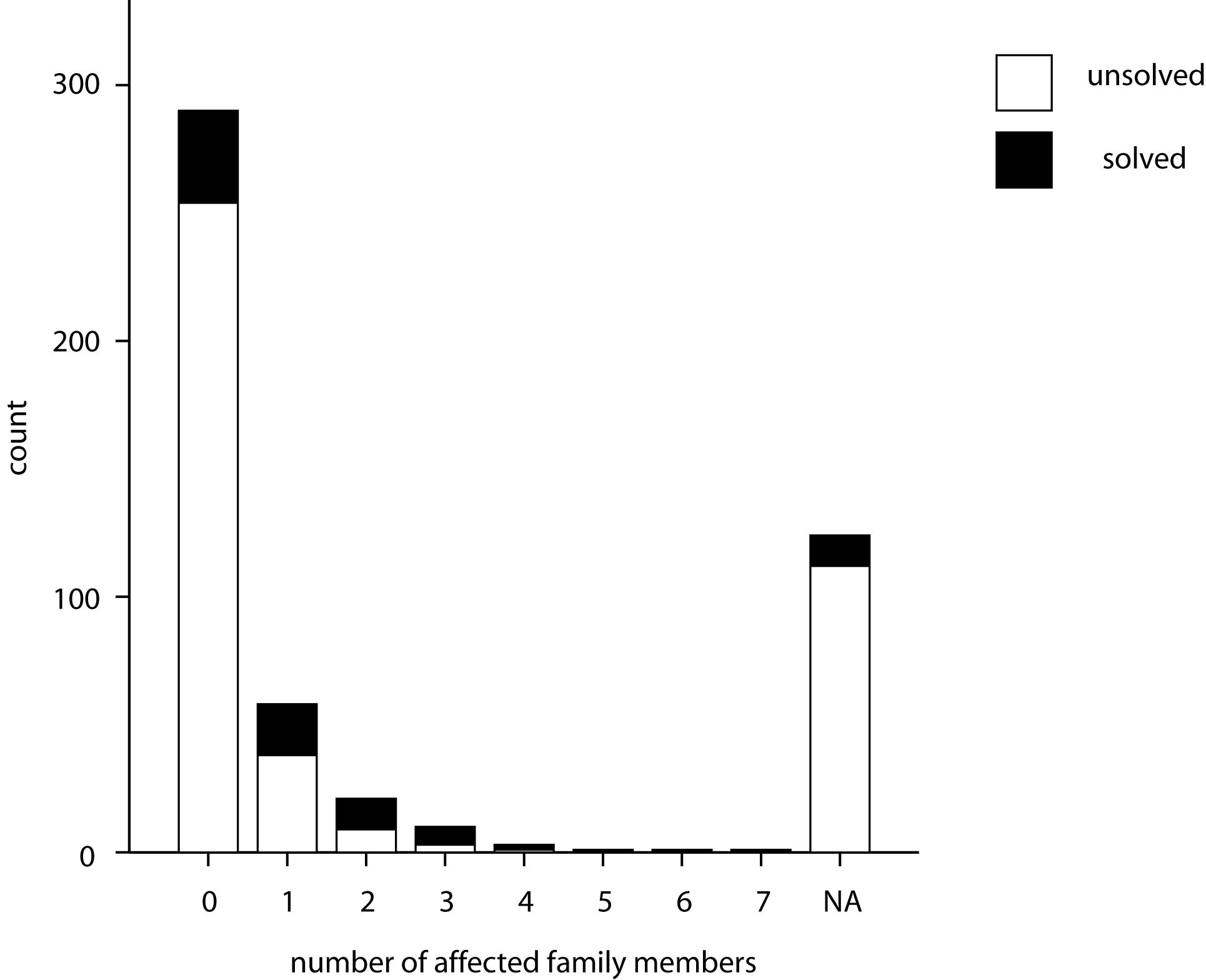
**

**Supplementary Figure 1: Number of genetically solved cases by the number of affected family members.** The number of affected family members is indicated on the x-axis, whereas NA indicates that no information on the family history was available. The number of individuals in which a disease causing variant could be identified is depicted in orange and the unsolved cases is indicated in blue.

**
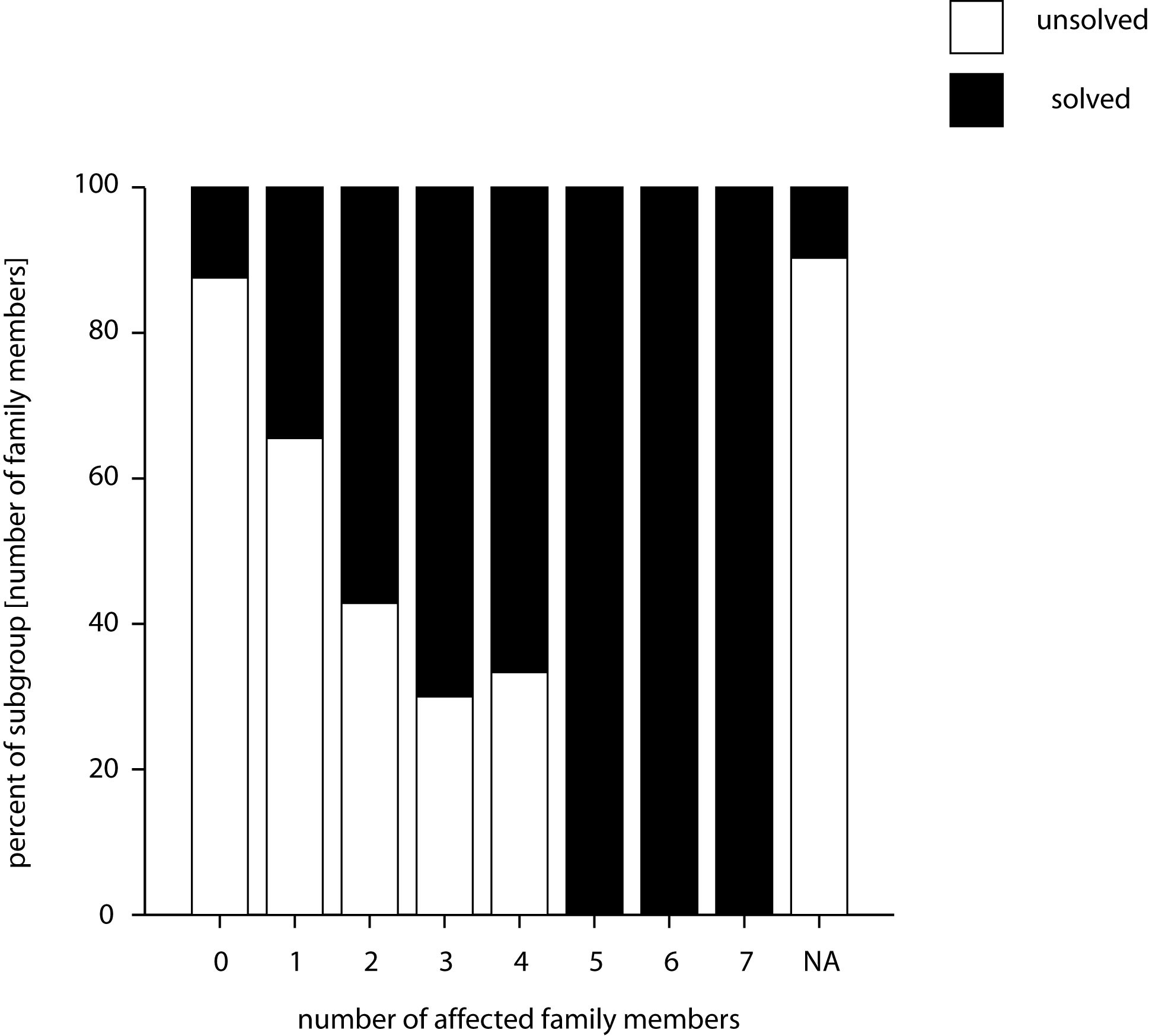
**

**Supplementary Figure 2: Proportion of genetically solved cases by the number of affected family members.** The number of affected family members is indicated on the x-axis, whereas NA indicates that no information on the family history was available. The percentage of individuals in which a disease causing variant could be identified is depicted in dark blue and the percentage of unsolved cases is indicated in light blue.

**
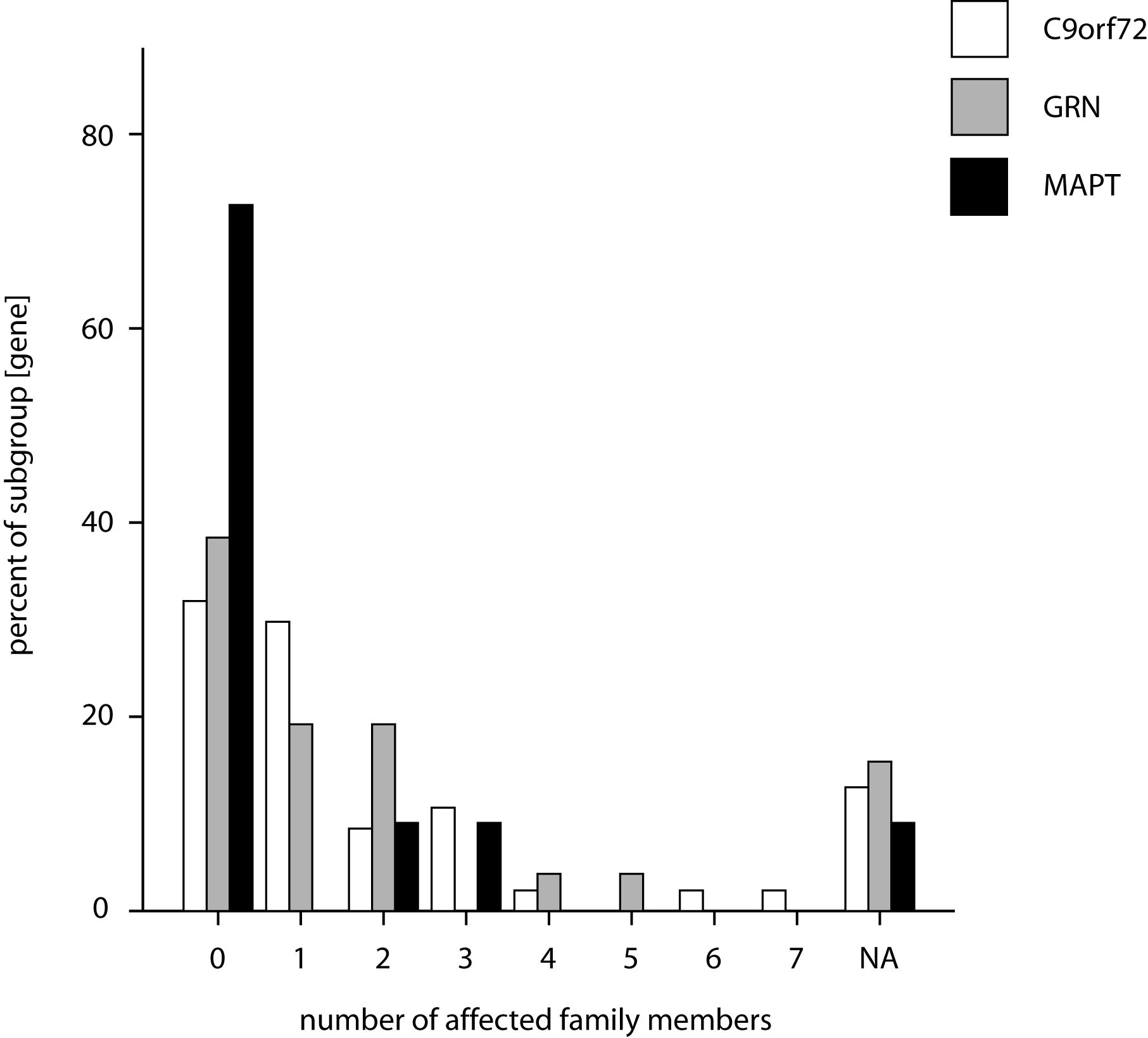
**

**Supplementary Figure 3: Distribution of the number of affected family members in cases with *C9orf72*, *GRN* or *MAPT* mutations.** The chart displays the proportion of the number of affected family members. No genetic diagnosis had a significantly different distribution of the numbers of affected family members.

**
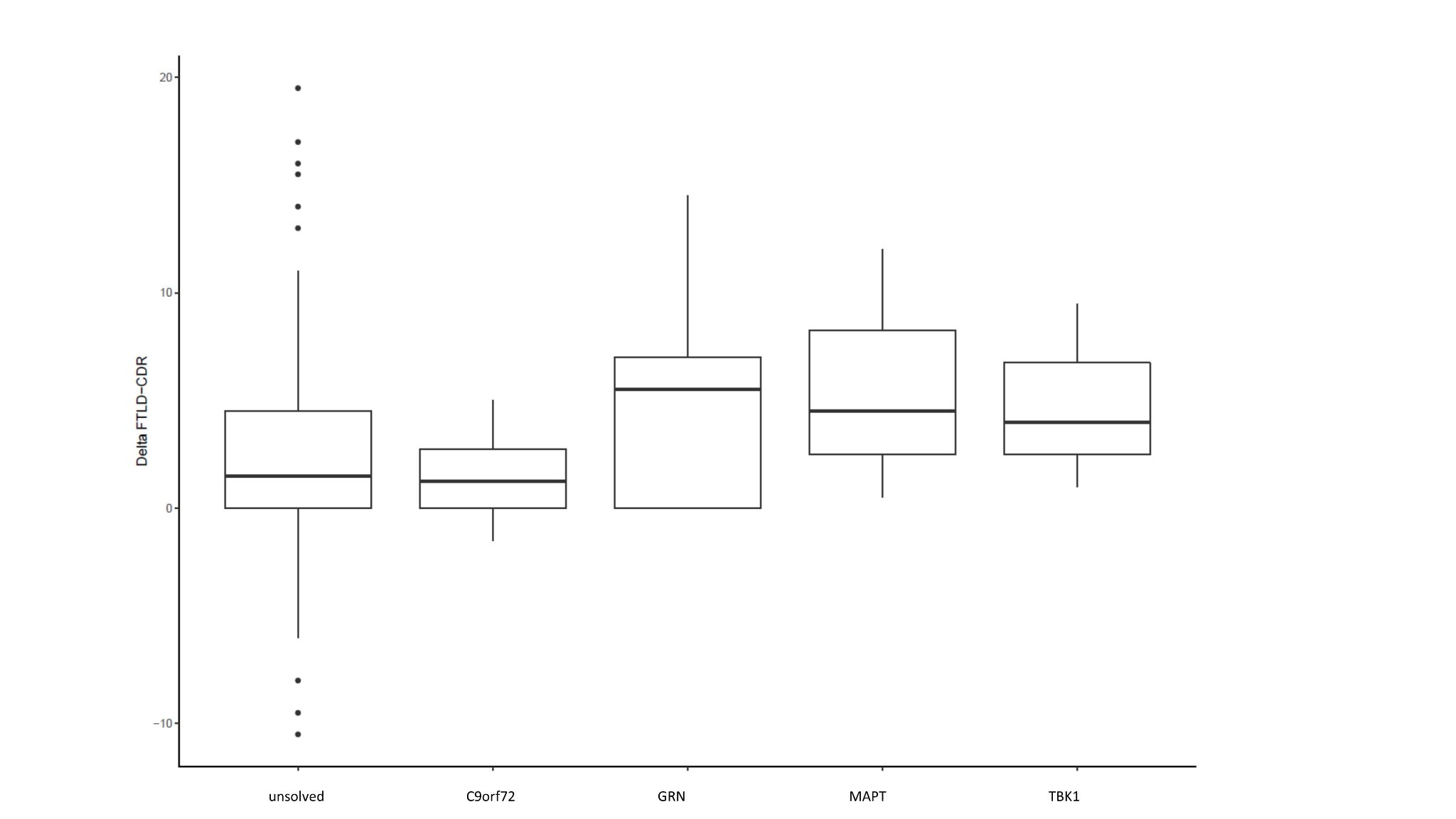
**

**Supplementary Figure 4: Delta-FTLD-CDR score distribution in the genetic subgroups.** To assess the rate of decline, we calculated the difference between the FTLD-CDR score at the first visit and at a year later (Delta-FTLD-CDR) for 156 individuals for which respective data was available. A high Delta-FTLD-CDR indicates a faster rate of decline whereas a negative Delta-FTLD-CDR indicates an improvement. We did not observe significant differences between the genetic subgroups (P = 0.11, Kruskal-Wallis).

**
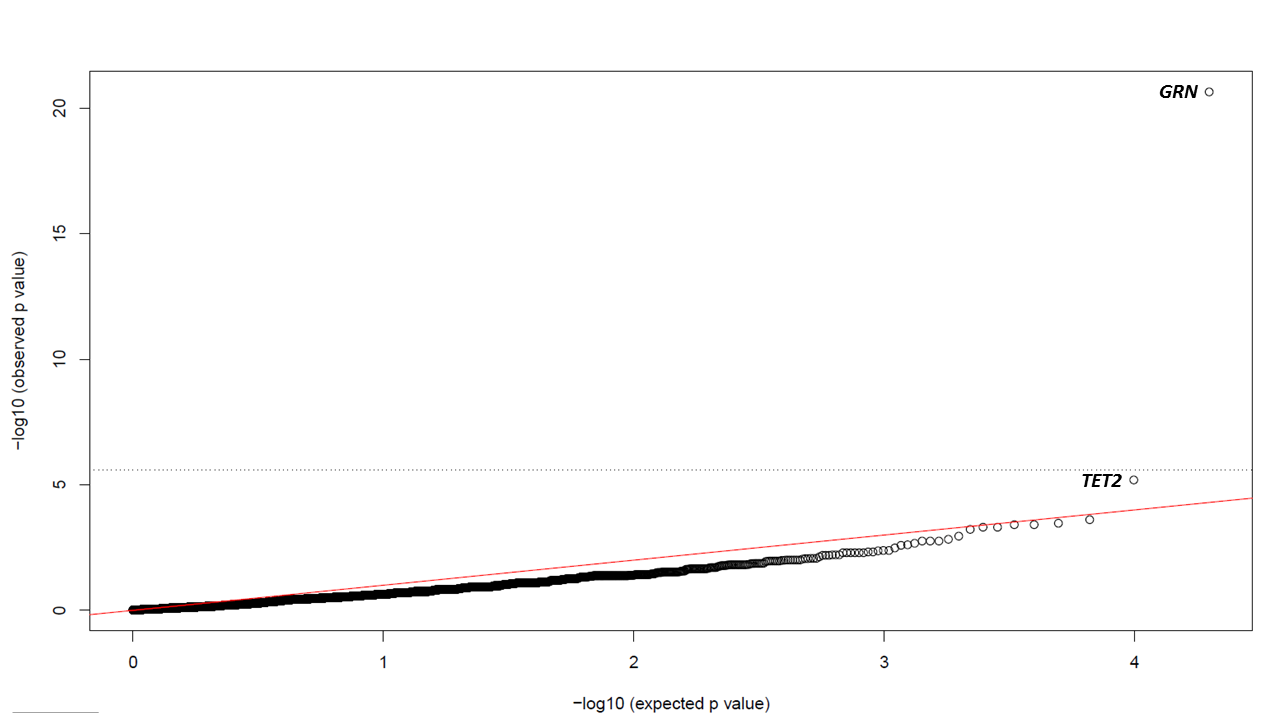
**

**Supplementary Figure 5: QQ plot of p-values from the burden analysis.** The QQ plot shows the observed plotted against the expected negative decadic logarithm of the P values from burden testing results for rare coding loss of function variants (minor allele frequency ≤ 0.1%) in 20,000 genes. The dotted line indicates the Bonferroni corrected p threshold. Besides an enrichment for variants in GRN (p = 2.2x10^-21^), a nominally significant enrichment for variants in TET2 (p= 6.4x10^-6^) was observed in the FTD cohort.

**Supplementary Tables**

**Supplementary Table 1:** Pathogenic and likely pathogenic variants identified.

| **Gene** | **CDS position** | **Genomic position** | **Protein change** | **Pathogenicity** |
| --- | --- | --- | --- | --- |
| ***GRN*** | **NM_002087.2** | **NP_002078.1** | **NC_000017.10** |  |
|  | c.103G>A | p.(Gly35Arg) | g.42426635G>A | pathogenic |
|  | c.146G>A | p.(Trp49Ter) | g.42426801G>A | pathogenic |
|  | c.264G>A | ? | g.42426919G>A | likely pathogenic |
|  | c.328C>T | p.(Arg110Ter) | g.42427098C>T | pathogenic |
|  | c.349+1G>C | ? | g.42427120G>C | pathogenic |
|  | c.424dup | p.(Met142AsnfsTer18) | g.42427669_42427670del | pathogenic |
|  | c.675_676del | p.Ser(226TrpfsTer28) | g.42428135_42428136del | pathogenic |
|  | c.675_676del | p.Ser(226TrpfsTer28) | g.42428135_42428136del | pathogenic |
|  | c.675_676del | p.Ser(226TrpfsTer28) | g.42428135_42428136del | pathogenic |
|  | c.675_676del | p.Ser(226TrpfsTer28) | g.42428135_42428136del | pathogenic |
|  | c.708+1G>A | ? | g.42428169G>A | pathogenic |
|  | c.709-2A>G | ? | g.42428403A>G | pathogenic |
|  | c.709-2A>G | ? | g.42428403A>G | pathogenic |
|  | c.709-2A>G | ? | g.42428403A>G | pathogenic |
|  | c.709-4_713del | ? | g.42428397_42428405del | pathogenic |
|  | c.759_760del | p.(Cys253Ter) | g.42428449_42428450del | pathogenic |
|  | c.759_760del | p.(Cys253Ter) | g.42428449_42428450del | pathogenic |
|  | c.759_760del | p.(Cys253Ter) | g.42428449_42428450del | pathogenic |
|  | c.759_760del | p.(Cys253Ter) | g.42428449_42428450del | pathogenic |
|  | c.882T>G | p.(Tyr294Ter) | g.42428777T>G | pathogenic |
|  | c.918C>A | p.(Cys306Ter) | g.42428813C>A | pathogenic |
|  | c.1252C>T | p.(Arg418Ter) | g.42429455C>T | pathogenic |
| ***MAPT*** | **NM_005910.5** | **NP_005901.2** | **NC_000017.10** |  |
|  | c.841_843del | p.(Lys281del) | g.44087691-44087693 | pathogenic |
|  | c.902C>T | p.(Pro301Leu) | g.44087755C>T | pathogenic |
|  | c.902C>T | p.(Pro301Leu) | g.44087755C>T | pathogenic |
|  | c.915+16C>T | ? | g.44087784C>T | pathogenic |
|  | c.915+16C>T | ? | g.44087784C>T | pathogenic |
|  | c.915+17G>A | ? | g.44087785G>A | likely pathogenic |
|  | c.915+17G>A | ? | g.44087785G>A | likely pathogenic |
|  | c.959C>T | p.(Ser320Phe) | g.44091652C>T | pathogenic |
|  | c.1008G>C | p.(Gln336His) | g.44095994G>C | pathogenic |
|  | c.1008G>C | p.(Gln336His) | g.44095994G>C | pathogenic |
|  | c.1090C>A | p.(Pro364Thr) | g.44096076C>A | likely pathogenic |
| ***TBK1*** | **NM_013254.3** | **NP_037386.1** | **NC_000012.10** |  |
|  | c.86dupA | p.(Lys30GlufsTer4) | g.64849734_64849735del | pathogenic |
|  | c.87G>A | ? | g.64849737G>A | likely pathogenic |
|  | c.427C>T | p.(Arg143Cys) | g.64860749C>T | pathogenic |
|  | c.992+1G>A | ? | g.64875802G>A | pathogenic |
|  | c.1928_1930del | p.(Glu643del) | g.64891001_64891003del | pathogenic |
|  | Deletion Exon 6-21 | ? |  | pathogenic |
| ***FUS*** | **NM_004960.3** | **NP_004951.1** | **NC_000016.10** |  |
|  | c.1394-2del | ? | g.31202282del | likely pathogenic |
| ***TARDBP*** | **NM_007375.3** | **NP_031401.1** | **NC_000001.10** |  |
|  | c.1147A>G | p.(Ile383Val) | g.11082613A>G | pathogenic |
| ***CTSF*** | **NM_003793.3** | **NP_003784.2** | **NC_000011.10** |  |
|  | c.1247T>C | p.(Ile416Thr) | g.66332103A>G | pathogenic |

**Supplementary Table 2:** P-values for the distribution of clinical diagnoses and the final genetic subgroup.

|  | ***C9ORF*** | ***GRN*** | ***MAPT*** | ***TBK*** | ***FUS*** | ***TARBP*** | ***CTSF*** |
| --- | --- | --- | --- | --- | --- | --- | --- |
| **not classified** | 0.5649 | 0.3664 | 0.674 | 0.3776 | 0.0108 | 0.6943 | 0.6943 |
| **bvFTD** | 0.0324 | 0.5837 | 0.0596 | 0.492 | 0.3672 | 0.2665 | 0.2665 |
| **FTD-ALS** | 0* | 0.471 | 0.8357 | 0.5233 | 0.7762 | 0.7762 | 0.7762 |
| **nvPPA** | 0.2031 | 0.1681 | 0.3773 | 0.2174 | 0.7921 | 0.7921 | 0.7921 |
| **nfvPPA** | 0.0385 | 0.4871 | 0.8227 | 0.5305 | 0.7222 | 0.7222 | 0.7222 |
| **svPPA** | 0.027 | 0.1822 | 0.2117 | 0.4027 | 0.7093 | 0.7093 | 0.7093 |
| **PPAnc** | 0.1175 | 0.4238 | 0.4657 | 0.6249 | 0.8276 | 0.8276 | 0.8276 |

This table P displays the P values for the distribution of clinical diagnoses and the final genetic subgroup. Note, that displayed values have not been corrected for multiple testing. *The only significant difference after multiple testing was an enrichment of cases with FTD-ALS in the subgroup with a pathogenic variant in *C9orf72* (p<0.001, Bonferoni corrected p< 0,0037).

**Supplementary Table 3:** Distribution of ApoE-Alleles in the FTD cohort and in gnomAD

|  | **ApoE2** | **ApoE3** | **ApoE4** |
| --- | --- | --- | --- |
| **FTD** | 6,88% | 78,78% | 14,34% |
| **published data** | 8,40% | 77,90% | 13,70% |
| **gnomAD** | 6,50% | 79,20% | 14,30% |

**Supplementary Table 4:** Rare loss-of-function variants in *TET2* identified in our FTD cohort (n=512).

| **ID** | **Age (years)** | **Variant in *TET2* (NM_001127208.2)** | **Coverage** | **Variant allele fraction (VAF)** | **COSMIC** | **gnomAD** |
| --- | --- | --- | --- | --- | --- | --- |
| 1 | 77 | c.2749C>T, p.(Gln917Ter) | 91 | 11% | COSM5487353 | not listed |
| 2 | 74 | c.3820C>T, p.(Gln1274Ter) | 103 | 13% | COSM96488 | not listed |
| 3 | 75 | c.4312A>T, p.(Lys1438Ter) | 108 | 11% | COSM4383946 | not listed |
| 4 | 60 | c.5329_5330del, p.(Leu1777SerfsTer11) | 65 | 9% | not listed | not listed |
| 5 | 73 | c.5476del, p.(Glu1826LysfsTer7) | 113 | 41% | not listed | not listed |
| 6 | 60 | c.5554C>T, p.(Gln1852Ter) | 135 | 13% | COSM42076 | not listed |
| 7 | 70 | c.1273del, p.(Thr425LeufsTer2) | 128 | 18% | not listed | not listed |
| 8 | 72 | c.1273del, p.(Thr425LeufsTer2) | 134 | 13% | not listed | not listed |

**Supplementary Table 5:** Rare loss-of-function variants in *TET2* identified in our control cohort (n=12 126).

| **ID** | **Age (years)** | **Variant in *TET2* (NM_001127208.2)** | **Coverage** | **Variant allele fraction (VAF)** | **COSMIC** | **gnomAD** |
| --- | --- | --- | --- | --- | --- | --- |
| 1 | unknown | c.4021dup, p.(Ala1341GlyfsTer3) | 118 | 36% | not listed | not listed |
| 2 | unknown | c.1692G>A, p.(Trp564Ter) | 281 | 25% | COSM7338431 | not listed |
| 3 | unknown | c.3500+2T>C, p.(?) | 122 | 15% | COSM87119 | not listed |
| 4 | 25 | c.2827C>T, p.(Gln943Ter) | 118 | 30% | COSM43467 | listed 1x |
| 5 | unknown | c.2674C>T, p.(Gln892Ter) | 58 | 19% | COSM6023715 | not listed |
| 6 | unknown | c.1912_1916del, p.(Met638SerfsTer41) | 64 | 20% | not listed | not listed |
| 7 | unknown | c.1566del, p.(Ser522ArgfsTer11) | 93 | 23% | not listed | not listed |
| 8 | 18 | c.1975C>T, p.(Gln659Ter) | 181 | 22% | COSM6498497 | not listed |
| 9 | unknown | c.5824C>T, p.(Gln1942Ter) | 160 | 31% | COSM4383975 | not listed |
| 10 | unknown | c.5182G>T, p.(Glu1728Ter) | 106 | 24% | not listed | not listed |
| 11 | 45 | c.2926C>T, p.(Gln976Ter) | 186 | 27% | COSM1738136 | not listed |
| 12 | unknown | c.4044+1G>A, p.(?) | 110 | 14% | COSM5945739 | not listed |
| 13 | unknown | c.4899_4903del, p.(Cys1633TrpfsTer26) | 320 | 24% | not listed | not listed |
| 14 | 15 | c.3737C>A, p.(Ser1246Ter) | 219 | 29% | COSM1168024 | not listed |
| 15 | unknown | c.5486del, p.(Pro1829HisfsTer4) | 119 | 16% | COSM110746 | not listed |
| 16 | unknown | c.1051G>T, p.(Glu351Ter) | 103 | 19% | COSM5708953 | not listed |
| 17 | unknown | c.2629_2630ins, p.(Asp877ValfsTer24) | 68 | 13% | not listed | not listed |
| 18 | unknown | c.4757C>A, p.(Ser1586Ter) | 351 | 31% | COSM110784 | not listed |
